# Study protocol – A Randomized controlled trial of efficacy of metronome on quality of chest compressions during simulated cardiopulmonary resuscitation among novice nurses

**DOI:** 10.1016/j.mex.2026.103818

**Published:** 2026-02-09

**Authors:** Mohanraj Harikrishnan, Eswari Solayappan, Shreedevi Gandhamaneni, Renuka MK, Ravishankar Nagaraja, Sharon Sheeba T, Ramesh Chandrababu

**Affiliations:** a Nurse practitioner, Department of Critical Care Medicine, Sri Ramachandra Institute of Higher Education and Research (Deemed to be University), Porur, Chennai 600116, India; b Research Associate III, Technical Resource Centre, Centre for Evidence Based Guidelines, Sri Ramachandra Institute of Higher Education and Research (Deemed to be University), Porur, Chennai 600116, India; c Senior Consultant, Department of Emergency Medicine, Sri Ramachandra Medical Centre, Sri Ramachandra Institute of Higher Education and Research (Deemed to be University), Porur, Chennai 600116, India; d Professor and Head, Department of Critical Care Medicine, Sri Ramachandra Medical College and Research Institute, Sri Ramachandra Institute of Higher Education and Research (Deemed to be University), Porur, Chennai 600116, India; e Assistant Professor, Dept. of Biostatistics, Vallabhbhai Patel Chest Institute Delhi, University of Delhi, Delhi 110007, India; f Professor and Head, Department of Medical-Surgical Nursing, Sri Ramachandra Faculty of Nursing, Sri Ramachandra Institute of Higher Education and Research (Deemed to be University), Porur, Chennai 600116, India

**Keywords:** Cardiopulmonary resuscitation, Metronome, Simulation, Healthcare, Wellbeing

## Abstract

**Background:**

High-quality chest compressions are essential for effective cardiopulmonary resuscitation (CPR), but novice nurses often struggle with maintaining the right compression rate, depth, and recoil. Simple tools like metronomes can help improve cardiopulmonary resuscitation quality by providing a steady rhythm for better performance.

**Objective:**

The objective is to evaluate whether metronome-guided CPR improves the quality of chest compressions performed by novice nurses in a simulated setting

**Method:**

This single-centric study of a simulation-based randomized controlled trial consists of 160 novice nurses divided equally into intervention and control groups (80 each).

Both groups performed a pre-test consisting of a 2-minute compression-only CPR session on a Q-CPR manikin without metronome guidance. After a 20-minute rest interval to prevent fatigue, the intervention group performed another 2-minute compression-only CPR session using a metronome set at 110 compressions per minute delivered. The control group repeated the CPR session under standard conditions without metronome assistance.

**Expected outcome:**

This study hypothesizes that novice nurses using a metronome throughout simulated cardiopulmonary resuscitation will achieve significantly higher quality chest compressions compared to those who do not use a metronome. It is expected that metronome-guided cardiopulmonary resuscitation will enhance the accuracy, depth, and consistency of compressions.

## Graphical abstract

**Figure:**
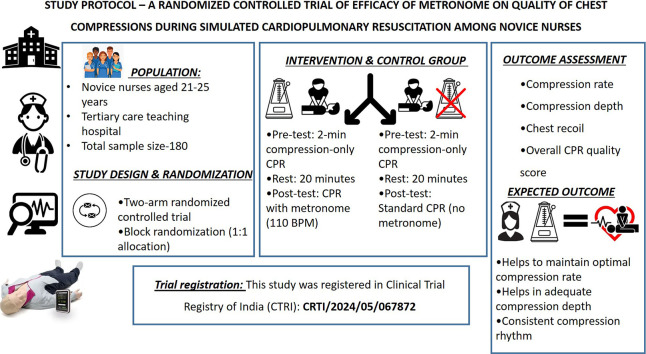

## Specifications table

**Table:** 

**Subject area**	Medicine and Nursing
**More specific subject area**	Nursing education, Critical care
**Name of your protocol**	Metronome assisted CPR training for community based nursing education
**Reagents/tools**	• Q-CPR Manikin (used to simulate adult CPR) • Resusci Anne wireless skill reporter software (Integrated with the manikin • Metronome (provided auditory guidance during chest compressions) • Personal computer/ Laptop (To run the skill reporter software)
**Experimental design**	Randomized controlled trials (block randomization) with 1:1 allocation between intervention and control groups. **Intervention group:** Participants allocated to the intervention group underwent **metronome-guided cardiopulmonary resuscitation (CPR)**. Initially, all participants completed a **baseline (pre-test)** consisting of a 2-minute session of compression-only CPR on a Q-CPR manikin without any auditory guidance. Following a standardized **20-minute rest period** to minimize fatigue effects, participants in the intervention group performed a **post-test 2-minute compression-only CPR session** with **metronome assistance set at 110 beats per minute (BPM),** delivered via a mobile phone application. **Control group:** Participants in the control group followed the same **study timeline and testing protocol** as the intervention group, ensuring methodological consistency. They completed a **2-minute pre-test compression-only CPR session** on the Q-CPR manikin without metronome guidance, followed by a **20-minute rest interval**. During the **post-test,** control group participants performed another **2-minute compression-only CPR session under standard conditions,** without any auditory or feedback assistance.
**Trial registration**	This study was registered in Clinical Trial Registry of India (CTRI). The trial registration number is: CRTI/2024/05/067872.
**Ethics**	This study was conducted at the Simulation Lab of a tertiary care centre of the Sri Ramachandra Institute of Higher Education and Research (Deemed to be university) hospital following the Declaration of Helsinki and was approved by the institutional review board of the same institution (reference code: CSP/23/AUG/134/754, dated October 16, 2023). Written informed consent was obtained from the participants.
**Value of the Protocol**	Ensures **methodological rigor and transparency** by clearly defining standardized procedures for intervention delivery, outcome assessment, and data analysis, thereby minimizing bias and enhancing reproducibility. Enables **objective evaluation of metronome-guided CPR** by systematically comparing intervention and control groups under identical simulated conditions. Provides a **replicable and scalable framework** for CPR training research, supporting evidence-based integration of low-cost tools like metronomes into nursing education and clinical practice.

## Background

Cardiac arrest, a critical medical emergency, necessitates an immediate and efficacious intervention to enhance the likelihood of survival. Cardiopulmonary resuscitation (CPR), an emergency medical technique, involves healthcare professionals administering chest compressions and artificial respirations to manually sustain cerebral function until advanced interventions can be implemented to re-establish spontaneous circulation and respiration in a patient experiencing cardiac arrest [1]. Consequently, medical personnel, encompassing physicians, nurses, and paramedics, must possess the capability to execute cardiopulmonary resuscitation as a core competency [2]. Nurses, as primary healthcare professionals, are crucial first responders; therefore, their expertise, abilities, and assurance in cardiopulmonary resuscitation are vital for preserving life [3]. Current guidelines stipulate that high-quality chest compressions are characterized by an optimal frequency of 100–120 compressions per minute (CPM), a compression depth of 50–60 mm, and complete chest recoil following each compression. This approach to chest compressions facilitates the most effective perfusion pressure and increases the probability of spontaneous circulation restoration [4].

Conversely, the attainment of a standardized and efficacious cardiopulmonary resuscitation (CPR) application presents a persistent difficulty, stemming from variations in technique, disparities in the experience of providers, and the inherently high-stress environment of resuscitation situations, even among healthcare professionals [5]. Furthermore, factors including fatigue, insufficient training, and emotional strain can adversely affect CPR performance, potentially influencing patient outcomes. To mitigate these obstacles and to augment CPR competency, novel pedagogical approaches, such as simulation-based training, have garnered increasing attention for their efficacy in improving nursing students' skills and fostering knowledge retention [6,7].

Metronome guidance offers a straightforward and cost-effective approach to enhancing cardiopulmonary resuscitation (CPR) techniques. This method delivers a consistent auditory cue, producing a steady beat at a specific tempo, quantified in beats per minute (BPM). Musicians frequently employ metronomes to maintain a uniform rhythmic pattern during performance [8]. In a comparable manner, a metronome can be programmed to the pre-established compression rate, serving as a reference point to assist rescuers in maintaining a consistent rhythm and pace throughout chest compressions. To our knowledge, this investigation represents the initial application of metronome-guided CPR training for novice nurses within a tertiary care hospital setting. Consequently, the study holds the potential to yield significant insights regarding the influence of metronome guidance on the quality of chest compressions during simulated CPR scenarios. The objectives of the study were to

• To assess the quality of chest compression during simulated cardiopulmonary resuscitation among novice nurses.
• To evaluate the efficacy of the metronome on the quality of chest compression during simulated cardiopulmonary resuscitation among novice nurses.

## Description of protocol

### Study design

This was an open-label, two-arm, randomized controlled, single-center study performed in the Simulation Lab of a tertiary care university teaching hospital from November 2023 to March 2024. The trial protocol for this investigation was registered prospectively on May 24, 2024, with the Clinical Trial Registry of India (CTRI) under the registration number CTRI/2024/05/067872. Additionally, approval was secured from the Institutional Ethics Committee of Sri Ramachandra Institute of Higher Education and Research (Deemed to be University), Chennai (reference code: CSP/23/AUG/134/754, dated October 16, 2023), and all procedures conformed to the principles outlined in the Declaration of Helsinki. All participants provided informed and written permission.

### Study settings, participants, and recruitment

The novice nurses were recruited in a tertiary care hospital of a multidisciplinary university. Before completing the written consent form, participants who satisfied the eligibility requirements obtained a thorough explanation of the study. Participants' eligibility criteria were as follows:

#### Inclusion criteria

• Novice nurses aged within 25 years.
• Novice nurses with basic cardiopulmonary resuscitation training.
• Those who are physically capable of performing chest compressions as per cardiopulmonary resuscitation guidelines.
• Novice nurses who have experience of less than 1 year.

#### Exclusion criteria

• Physicians, residents, and other healthcare professionals other than nurses were excluded.
• Those with medical conditions or physical limitations that may prevent them from safely performing chest compressions (musculoskeletal disorders, cardiovascular conditions, neurological deficits, pregnancy).
• Participants who fail to provide informed consent or withdraw from the study before completion.

### Sample size

Referring to the previous study, Use of a Metronome in CPR: A Simulation Study [9], the estimated sample size was determined to be 160 participants; this would achieve the power of 80% at a level of significance of 0.05. The calculated sample size per group is 72. Accounting for a 10% dropout rate, the adjusted sample size is 80 participants per group, resulting in a total of 160 participants in both the intervention and the control group. The Minimal Clinically Important Difference (MCID) of 22 compressions per minute for compression rate was derived from previous studies that evaluated performance variability during CPR training between the experimental group and the control group. Prior research using simulation-based assessments identified a change of approximately 20–25 compressions per minute as the threshold at which differences in performance become clinically relevant and perceptible in terms of resuscitation quality [10,11]

### Randomisation, allocation concealment, and blinding

**Fig. 1:**
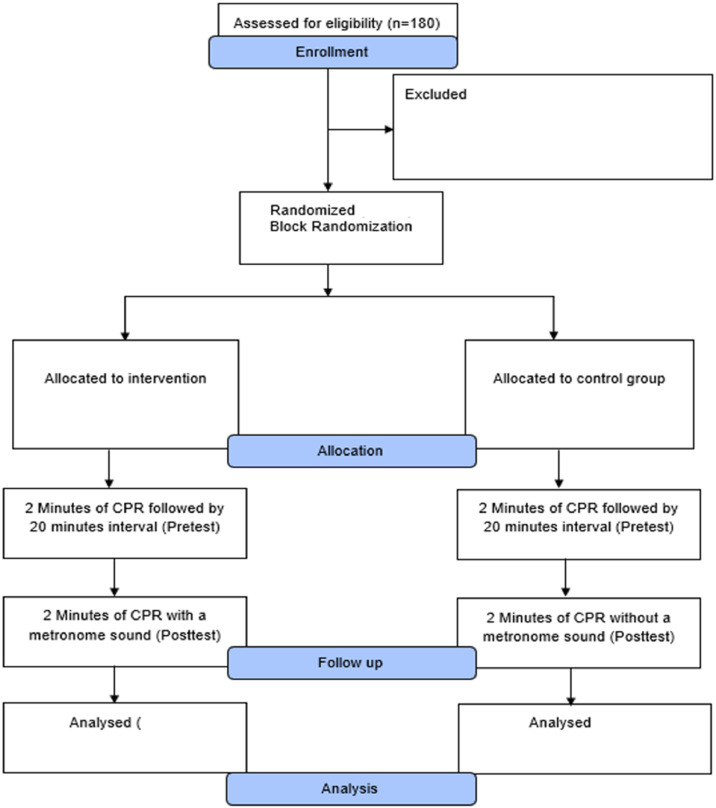
CONSORT flowchart illustrating the stages of participant recruitment, allocation, follow-up, and analysis in the randomized controlled trial.

A total of 180 participants were assessed for eligibility, and 20 were excluded (12 did not meet the inclusion criteria, and 8 declined to participate). The remaining 160 participants were enrolled in the study. The flow of participants through the study is presented in Fig. 1 (CONSORT flow diagram). A block randomization was utilized to ensure balanced allocation of participants between the intervention and control groups. The total sample was separated into 16 blocks, with each block containing 10 participants—5 assigned to the study group (metronome-assisted CPR) and 5 to the control group (standard CPR without metronome guidance). This approach was chosen to maintain equal group sizes throughout the enrollment process and to minimize potential biases.

Allocation concealment was achieved using a sealed envelope block randomization method. Opaque, sequentially numbered envelopes containing the group assignments were prepared in advance and mixed thoroughly. As each participant enrolled in the study, they were asked to select a sealed envelope, which determined their group allocation. This method ensured that neither the participants nor the researchers could predict group assignments prior to randomization, thus reducing selection bias.

### Intervention and data collection

Following randomization, all participants did a 2-minute pretest of compression-only cardiopulmonary resuscitation on a Q-CPR manikin without any metronome guidance. This pretest was conducted to establish a baseline measurement of chest compression quality for both groups under identical conditions. Data for each participant was recorded using the Q-CPR manikin, connected to the Resusci Anne Wireless Skill Reporter software and a personal computer skill reporting system (Laerdal Medical AS, Stavanger, Norway). After a 20-minute rest interval, to prevent fatigue from affecting post-test performance, the intervention group performed a 2-minute compression-only CPR session on the QCPR manikin, this time guided by a metronome set to 110 beats per minute (BPM), delivered through a mobile phone application. In contrast, the control group completed the post-test without any metronome assistance, replicating standard CPR practice.

The system measured key CPR quality indicators, focusing on three core components: adequate chest compression depth, compression rate, and full chest recoil. In addition to these specific metrics, the software generated an overall performance score based on each participant’s adherence to CPR guidelines. This ensured an accurate and objective assessment of CPR quality, providing consistent data for evaluating the impact of metronome guidance on chest compression performance.

To ensure objective evaluation, both groups underwent a post-test assessment measuring key CPR performance indicators, including compression rate, depth, and recoil. While full blinding of participants was not feasible due to the audible metronome in the study group, efforts were made to blind the data analysts to group assignments during the evaluation phase to minimize assessment bias.

### Outcome measures

The primary outcome measures focused on evaluating the quality of chest compressions performed during cardiopulmonary resuscitation (CPR). These included:

Compression Rate: Measured in compressions per minute (cpm), with an optimal range defined as 100–120 cpm according to American Heart Association (AHA) guidelines.

Compression Depth: Recorded in millimeters, assessing whether compressions met the recommended depth of 51–61 mm.

Chest Recoil: Evaluated based on the proportion of compressions allowing complete chest recoil, important for effective blood flow during CPR.

Overall CPR Quality Score: The overall CPR quality score was automatically generated by the Q-CPR skill reporter system integrated with the Resusci Anne manikin. The software calculates a composite score (ranging from 0 to 0–100%) based on real-time assessment of four key parameters: compression rate, compression depth, recoil, and hand position accuracy. Each parameter contributes proportionally to the total quality score according to the system’s internal algorithm, which follows the American Heart Association (AHA) 2020 guidelines for high-quality CPR. Thresholds for performance classification were defined by the Q-CPR software as follows:

• Good performance: Overall quality score ≥ 95%
• Average performance: Overall quality score between 85%–94%
• Poor performance: Overall quality score < 84%

These thresholds are automatically applied by the Q-CPR software to categorize the rescuer’s performance after each simulation session. The score thus represents an integrated measure of compression quality that reflects adherence to recommended CPR parameters.

Secondary outcome measures included:

• The impact of metronome guidance on maintaining optimal compression rhythm.
• The difference in CPR performance between pretest and post-test phases within and between groups.

These outcome measures were chosen to comprehensively assess the effectiveness of metronome guidance in enhancing CPR quality among novice nurses.

### Data analysis

The data analysis was conducted using SPSS version 22, employing both descriptive and inferential statistics to address the study objectives and test the hypotheses. Descriptive statistics, including frequency, percentage, mean, and standard deviation, will be used to summarize demographic variables and baseline characteristics. Inferential statistical analyses will be applied to evaluate the impact of metronome-guided CPR on novice nurses. Categorical comparisons will not be done, as the samples are almost similar age groups. Repeated measures ANOVA will be utilized to assess within-group and between-group differences over time in key CPR quality indicators such as compression rate, depth, recoil, and overall performance. A chi-square test will be performed for the post-test. Paired t-tests will be conducted to compare pretest and post-test scores within each group, while independent t-tests will assess differences between the intervention and control groups in the post-test phase. The level of statistical significance was set at *p* < 0.05. Results will be presented using tables and graphs to facilitate clarity and interpretation.

## Protocol validation

Novice nurses, who have limited clinical experience, often face challenges in delivering high-quality CPR, particularly in maintaining optimal compression rate, depth, and recoil. Targeted training and supportive tools, such as metronome-guided feedback, are essential to enhance CPR performance, ensuring that novice nurses can provide effective and consistent resuscitation in critical situations. Although studies have been conducted on nurses, there have been no studies on novice nurses, and this will be the first study in the previous scientific literature to look particularly at the effect of metronome-guided CPR on the quality of chest compressions done by novice nurses. A study by Rikhotso et al. indicated that despite nurses’ prior resuscitation training, years of experience, and confidence in CPR, there was a lack of awareness and limited understanding of CPR, which is a vital skill that directly impacts patient survival during cardiac arrest [9,12]. A study conducted in Kuwait found that many critical care nurses lack CPR expertise and understanding [13].

A systematic review conducted in 2021 highlighted the impact of automated real-time feedback systems on chest compression performance during simulated CPR. The review found that regardless of the design—whether metronome [14,15], audio, visual, or multimedia—these systems led to a significant improvement in all tested parameters [[14], [15], [16], [17], [18], [19], [20]]. However, some studies reported improvements in certain parameters, such as compression rate and depth, while others, like leaning, remained unaffected. Two studies proved in another review that feedback devices enhance CPR skill acquisition, retention, and performance [21,22]. These findings underscore the potential benefits of feedback devices in enhancing CPR quality, though their effectiveness may vary across different compression metrics.

This study is novel as it concentrates on novice nurses and assesses the impact of a straightforward, economical, and easily scaled instrument, a metronome on the quality of chest compressions utilizing an objective, high-fidelity feedback system. This trial distinguishes itself from prior research by focusing solely on rhythmic auditory guidance as an intervention, employing a randomized controlled design with pre- and post-assessment to systematically evaluate its effects. The project integrates simulation-based training with real-time quantitative performance indicators, thereby bridging a significant gap between educational theory and practical skill acquisition for early-career nurses. The results could guide evidence-based adjustments to CPR training programs, especially in resource constrained environments where advanced feedback devices are unavailable, although metronome applications are readily accessible. We believe the study warrants publication due to its innovative, methodologically sound, and clinically pertinent contribution, illustrating how a straightforward intervention can markedly improve resuscitation quality, thus promoting safer practices, enhanced learning outcomes, and potentially increased patient survival in actual cardiac arrest situations.

Moreover, the use of simulation-based training offered a controlled environment where novice nurses could perform CPR without the unpredictability of real-life clinical scenarios. This ensured that external variables, such as patient-specific complexities or environmental stressors, did not influence the outcomes, allowing for a focused analysis on how metronome guidance alone affected CPR performance.

### Limitations

Although high-fidelity simulation offered a controlled setting, the results' generalizability may be limited since it might not accurately represent the intricacies and demands of actual cardiac arrest situations. The study's emphasis on short-term evaluation also prevented it from assessing long-term skill retention or whether gains would last in the absence of consistent metronome use. Lastly, the study only evaluated compression metrics (rate, depth, and recoil) without evaluating other crucial aspects of CPR that significantly affect resuscitation results, such as ventilation quality, hands-off time, or team dynamics.

## Supplementary material *and/or* additional information

Nil.

## Related research article

None.

## Financial interests/personal relationships

Nil.

## CRediT authorship contribution statement

**Mohanraj Harikrishnan:** Writing – original draft, Investigation. **Eswari Solayappan:** Writing – review & editing. **Shreedevi Gandhamaneni:** Data curation, Validation, Resources. **Renuka MK:** Investigation, Resources, Writing – review & editing. **Ravishankar Nagaraja:** Formal analysis. **Sharon Sheeba T:** Data curation, Software. **Ramesh Chandrababu:** Conceptualization, Methodology, Project administration, Supervision.

## Declaration of competing interest

The authors declare that they have no known competing financial interests or personal relationships that could have appeared to influence the work reported in this paper.

## Data Availability

No data was used for the research described in the article.
